# The Corrosion Susceptibility of 304L Stainless Steel Exposed to Crevice Environments

**DOI:** 10.3390/ma15093055

**Published:** 2022-04-22

**Authors:** Kun-Chao Tsai, Chun-Ping Yeh

**Affiliations:** Division of Nuclear Fuels and Materials, Institute of Nuclear Energy Research, 1000 Wenhua Rd., Longtan District, Taoyuan City 32546, Taiwan; cpyeh@iner.gov.tw

**Keywords:** 304L, stainless steel, crevice corrosion

## Abstract

The present study focuses on the corrosion behavior of 304L stainless steel in crevice corrosion environments. The specimen with a salt deposit of 0.1 g/m^2^ was assembled with a crevice former made of Poly-tetra fluoroethylene (PTFE) to make a test device. The assembled test devices were kept at the ambient temperature of 45 °C in combination with a relative humidity of 45%, 55%, and 70%. After testing for 5000 h, the corroded area of the specimen exposed to 70% humidity was three times larger than that subjected to 45% humidity. For the specimen sustaining a tensile force, the crack growth rate was approximately 1.4 mm/year at the stress level of 300 MPa in a crevice corrosion environment with 0.1 g/m^2^ of sea salt deposited on the surface. The small portion of intergranular cracking occurred at the surface due to the existed strain on the surface. As cracks propagate in a grain, the grain undergoes a greater localized deformation, and some secondary cracks would develop inside the grain; transgranular cracking was vigorous due to the path corrosion that nucleated at the slip steps.

## 1. Introduction

Crevice corrosion is well known to develop in narrow gaps, due to the stagnant liquid and oxygen in the crevice area, where intensive corrosion occurs in the localized area when electrolyte movement is restricted. The lack of oxygen inside the crevice confines the oxidation-reduction reaction to the exterior surface; anodic and cathodic reactions occur, respectively, inside the crevice and other bulk areas [1,2,3,4,5]. 

The corrosion susceptibility of austenitic stainless steels Increases with increasing the chloride concentrations. In the corrosion pit or crevice, the surrounding electrolyte gains positive electrical charges. Therefore, the positively charged pit attracts negative ions of chlorine. The chloride ions, from the ambient environment, accumulated on the surface, cause the corrosion rate to increase. The chloride ions attack the passive films on the surface of stainless steel; their reaction with the metal matrix causes the subsequent growth of stress corrosion cracking (SCC), which is an important issue for stainless steel installations on the seaboard [6]. In addition, cracks propagate and grow more quickly in the crevice filled with chloride. The initiation stage and the consequent stage of crevice corrosion are presented below [2,3,4,5]: 

Stage I:

M → M^2+^ + 2e^−^

O_2_ + 2H_2_O + 4e^−^ → 4OH^−^

Stage II:

M^2+^ + H_2_O → M(OH)_2_ + H^+^

2Cl^−^ + M^2+^ → MCl_2_

MCl_2_ + 2H_2_O → M(OH)_2_ + 2H^+^ + 2Cl^−^

In atmospheric environments, Tokiwai et al. [7] investigated the generation of SCC with a variation of the tensile stress level and relative humidity. The results showed that SCC occurred on the 304 stainless steel surface at the temperature of 50 °C and relative humidity of 70 to 98% with 8 mg/m^2^ of salt on the specimen’s surface. However, with a relative humidity of 60%, SCC can be found with only an amount of 55 mg/m^2^ of salt. Guo et al. [8] studied the relative humidity change in the context of atmospheric pitting corrosion; the results showed that relative humidity fluctuations lead to the nucleation of many small pits, which might help to prevent the growth of a large pit, whereas a constant 33% relative humidity leads to the growth of a single pit.

The studies indicate that the absorption of hydrogen increases the resistance to dislocation movement as well as the martensite formation, which functions as an obstacle to dislocation movement [9,10]. Austenite stainless steels suffered intergranular cracking at lower temperatures and transgranular cracking at higher temperatures [9,11,12]. At lower temperatures, the strain-induced formation of martensite tends to occur along the grain boundaries and is assisted by hydrogen [9,13,14,15]. The formation of martensite occurs at the grain boundary, which contributes to the intergranular cracking mechanism [9,15]. 

The martensite transformation and susceptibility to transgranular SCC is a result of the fact that transformation can occur by both the strain-induced mechanism and by the absorption of hydrogen into the austenite [10]. The diffusing hydrogen leads to the enhancement of the dislocation movement at the slip plane; the generation of dislocations on {111} slip planes and the subsequent brittle failure of these slip planes when the critical stress intensity is reached [9,12,14]. Transgranular cracking occurs by propagation cracks that nucleate at the slip steps by diffusion [9,15]. 

Many power plants in Taiwan are located in a coastal area, in a climate that is relatively hot and humid; dry cask storage systems are deployed in nuclear power plants for spent nuclear fuel storage when spent fuel pools reach their storage capacity. Consequently, a primary concern for the dry storage of spent nuclear fuel is chloride-induced stress corrosion cracking, caused by the deliquescence of salts deposited on the stainless steel canisters. Therefore, it is necessary to understand the corrosion mechanism of stainless steel in an environment with small amounts of sea salt, so that precautions can be taken to mitigate degradation. Turnbull et al. [16] examined the SCC on four-point samples with 5 to 10 µg/cm^2^ of MgCl_2_ on the specimens; the specimens were placed in a chamber at a relative humidity of 45% and temperature of 60 °C, which were assumed to simulate the expected salt concentrations on the exposed stainless steel sections of the nuclear plant located in coastal regions. Weirch et al. [17] studied the effect of relative humidity on the corrosion of 304 stainless steel; they used an inkjet printer with sea water; the printing produced a uniform field of discrete droplets with a salt loading density of 300 µg/cm^2^. In this work, the amounts of 0.1 g/m^2^, 1 g/m^2^, and 4 g/m^2^ of sea salt are used, owing to the deposited salt on the canister’s surface (inside the overpack) that was believed to be 0.1 g/m^2^, and 1 g/m^2^ for 19 years [18]. In light of the previous results [1,18], the specimen aging times of 1500 and 5000 h are chosen.

## 2. Experimental Procedures

**Figure 1:**
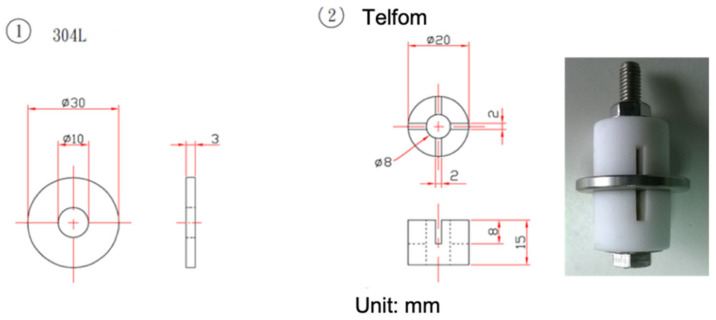
The dimensions of: (1) the SS 304L specimen and (2) crevice former device.

**Figure 2:**
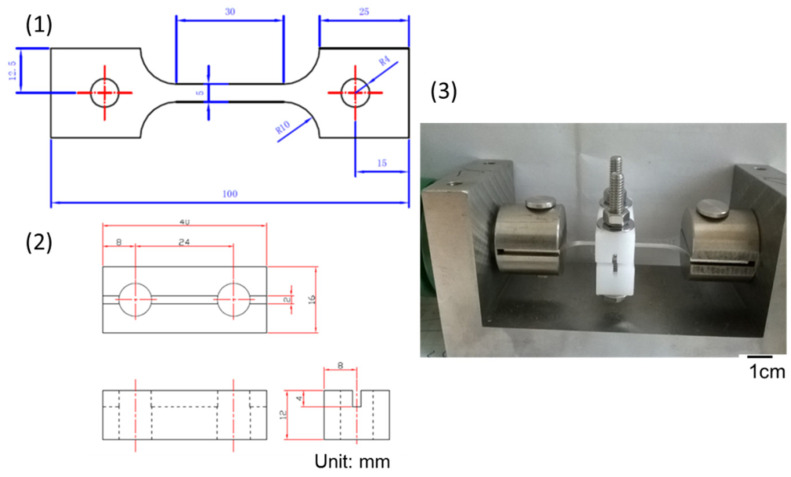
The dimensions of the SS 304L tensile test specimen and crevice former under tensile stress: (1) SS 304L specimen, (2) crevice former device, and (3) photo of the specimen set-up.

The compositions of commercial 304L (OLARRA S.A.) stainless steel plates are listed in Table 1. There were two kinds of specimens used in this work, which were mechanically polished with 2400-grit sandpaper and washed with distilled water. After the specimens’ surfaces were clean, they were firstly sprayed with sea salt mist, and the sea salt composite is revealed in Table 2. Then, the specimens were dried on a hot plate at the temperature of 60 °C for 10 min to leave residuals of 0.1 g/m^2^, 1 g/m^2^, and 4 g/m^2^ of salts on the specimens. Figure 1 and Figure 2 show the specimens’ dimensions and set-up of the crevice corrosion test and tensile test, respectively. Then, the crevice former of Poly-tetra fluoroethylene (PTFE) was fixed on the specimens’ surfaces with an M6 screw by applying a torque of 1.12 N⋅m (10 pound-inch) to form a crevice configuration. For the tensile test specimen, a jig was used to apply the load, as shown in Figure 2. The tensile stress loading apparatuses were acknowledged from the results published by Tokiwai et al. [6]. Before the specimen was set up on the jig, the tensile force was applied by a torque wrench and measured by a load cell at the end of the jig. The torque was recorded when the tensile force reached the scheduled stress. Accordingly, the tensile stress applied to the specimen was adjusted by using the torque wrench with the pre-set torque. The test environment chambers were kept at a combination of the ambient temperature of 45 °C and relative humidity of 45%, 55% a and 70%, respectively, for 400, 1500, and 5000 h each.

After the environmental tests, a cross-section of the corroded specimens was cut and polished, following the standard metallograph specimen preparation procedures, which were mechanically polished with 2400-grit sandpaper. The final polishing was conducted with a cloth-covered wheel, by adding alumina 0.05 µm suspension (Struers) and distilled water. Then, the metallographic specimens were examined with an optical microscope (OM, Olympus OLS4000) to characterize the cracks. A scanning electron microscope (SEM, Hitachi S-4800) was employed to study the surface morphology, which was also equipped with an energy dispersive X-ray spectroscope (EDS, Horiba) and electron beam backscattering diffraction (EBSD, EDAX) for further analysis. The kernel average misorientation (KAM) and grain average misorientation (GAM) were obtained from the orientation measurement via EBSD OIM analysis software.

## 3. Results and Discussion

### 3.1. The Crevice Corrosion Test

**Figure 3:**
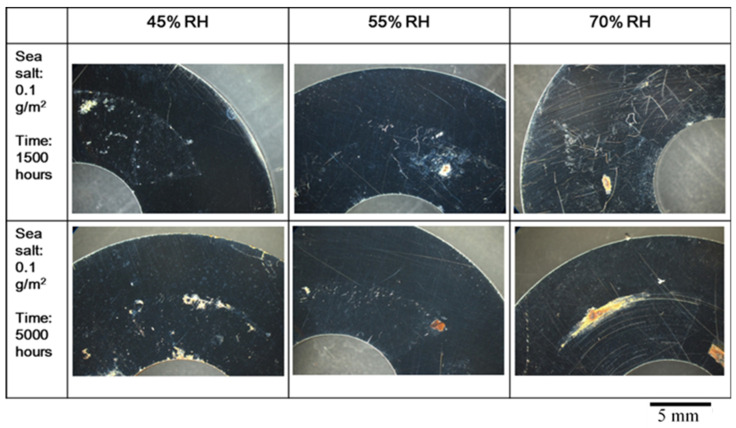
The specimens with 0.1 g/m^2^ of sea salt deposited on the surface were tested at different relative humidity levels and temperatures of 45 °C.

**Figure 4:**
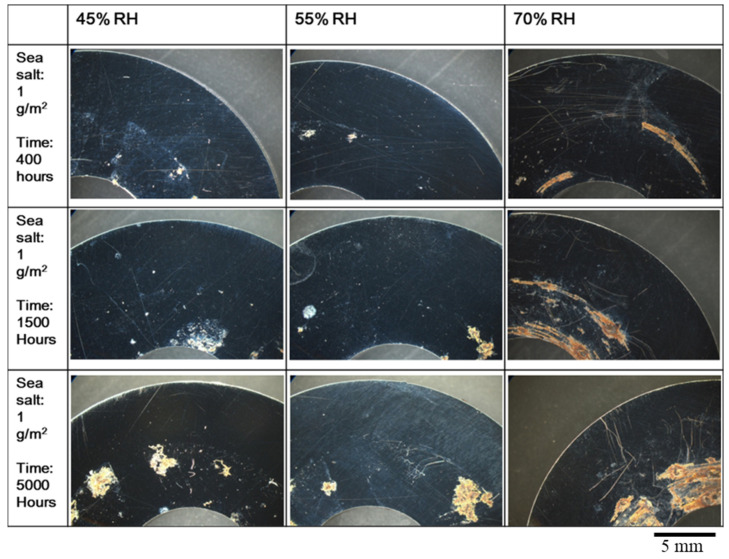
The round specimens with 1 g/m^2^ of sea salt deposited on the surface were tested at different relative humidity levels of 45%, 55%, and 70%, and a temperature of 45 °C.

Figure 3 and Figure 4 show the round specimens after testing at 45 °C in the crevice corrosive environments of different humidity levels for 1500 h and 5000 h. They were sprayed with 0.1 g/m^2^ and 1 g/m^2^ of sea salt onto their surface, respectively. The corrosion area increased with an increase in the relative humidity, the amount of salt, and test time. The increase in humidity will increase the wetting of the salt to attack the passive films of stainless steel. For the specimens with 0.1 g/m^2^ of sea salt on the surface, SCC was not perceived and the pitting corrosion mostly lead to the growth of a single pit.

**Figure 5:**
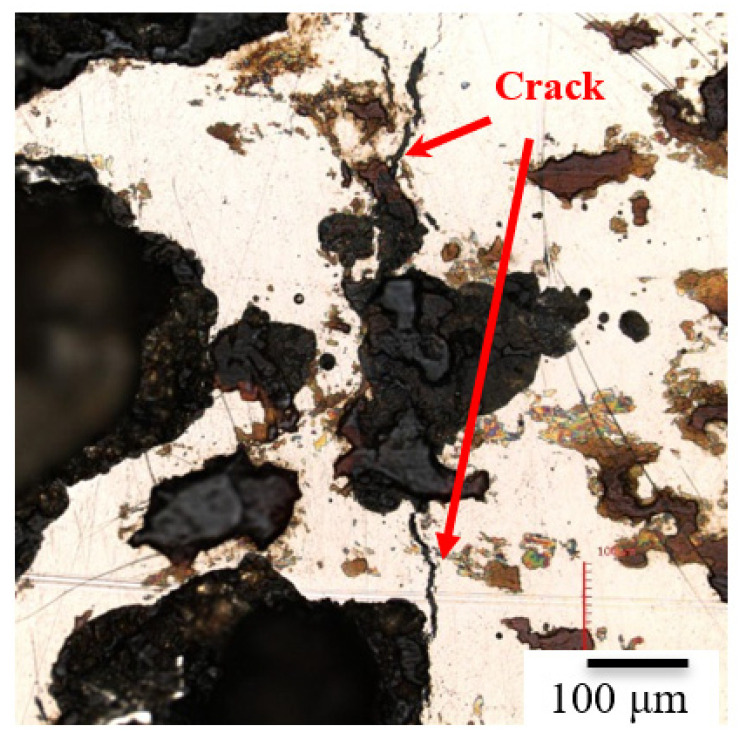
The round specimens show cracks after crevice corrosion testing at the relative humidity of 70% for 5000 h with 1 g/m^2^ of sea salt deposited on the surface.

The SCC developed after crevice tests for 5000 h, with which an amount of 1 g/m^2^ of sea salt was deposited on the specimen surface at a relative humidity of 70%, as shown in Figure 5. In addition, corrosion pits, as well as corrosion products, were observed beneath the former crevice; no notable corrosion pit or corrosion products were shown in the uncovered area. This suggests that the crevice increased the corrosion rate. For the corrosion specimens tested for 5000 h, the corroded area of the specimen in 70% humidity was 3 times higher than that in 45% humidity.

### 3.2. The Crevice Corrosion Test under Tensile Stress

**Figure 6:**
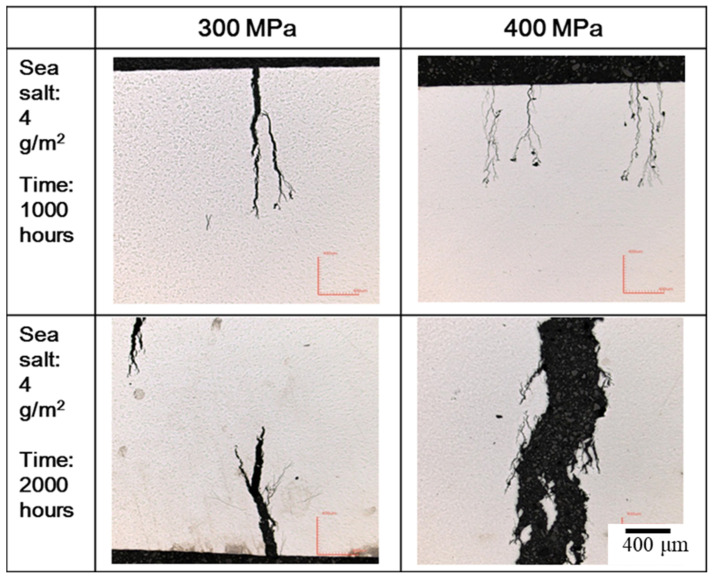
The crevice corrosion specimens with 4 g/m^2^ of sea salt deposited on the surface were subjected to tensile stresses of 300 MPa and 400 MPa at a temperature of 45 °C.

**Figure 7:**
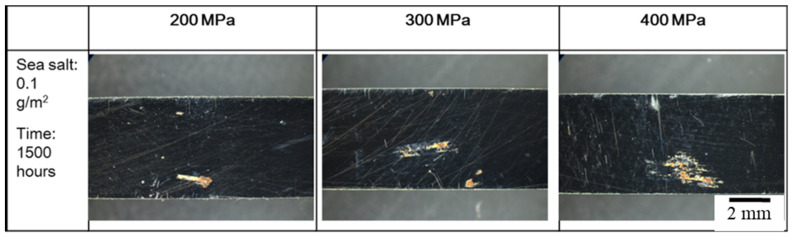
The crevice corrosion specimens with 0.1 g/m^2^ of sea salt deposited on the surface were subjected to tensile stresses of 200 MPa, 300 MPa, and 400 MPa at a temperature of 45 °C for 1500 h for each testing condition.

**Figure 8:**
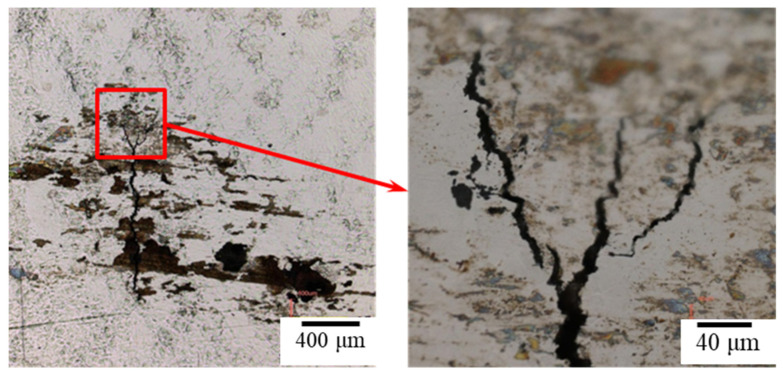
The crevice corrosion specimen with 0.1 g/m^2^ of sea salt deposited on the surface subjected to tensile stresses of 400 MPa reveals cracks after 1500 h of testing at 45 °C and a relative humidity of 45%.

**Figure 9:**
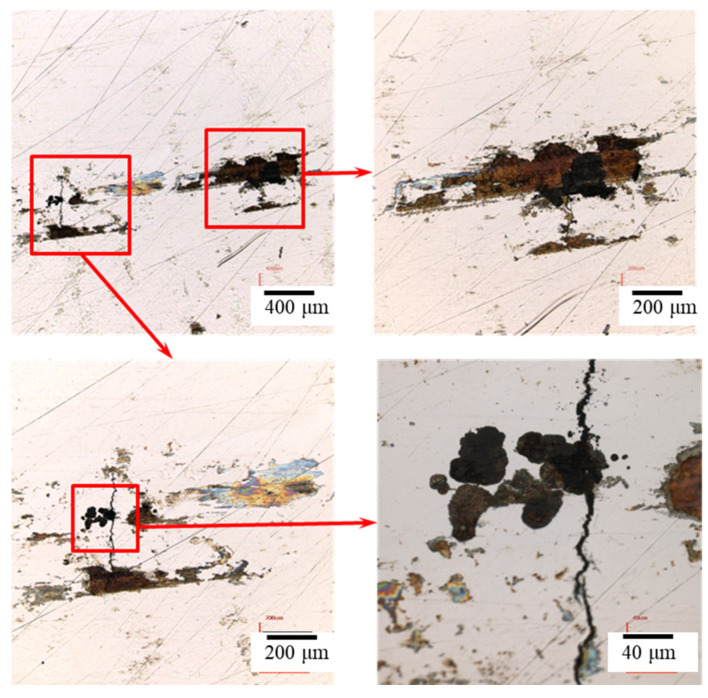
The crevice corrosion specimen with 0.1 g/m^2^ of sea salt deposited on the surface subjected to tensile stresses of 300 MPa reveals cracks after 1500 h of testing at 45 °C and a relative humidity of 45%.

**Figure 10:**
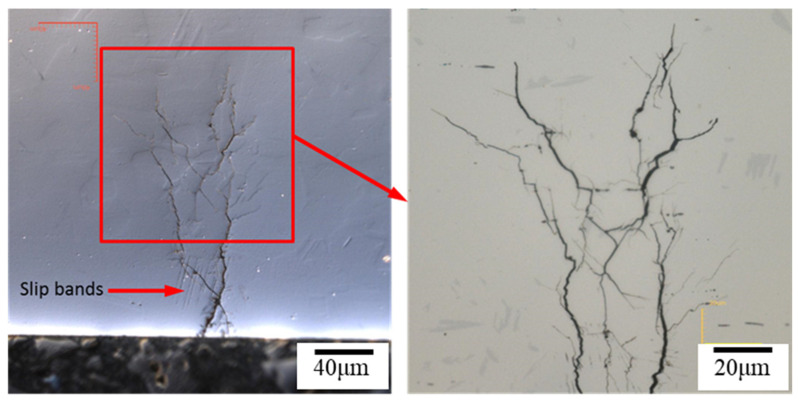
The cross-section of the specimen with 0.1 g/m^2^ of sea salt deposited on the surface reveals cracks after testing at 300 MPa tensile stress for 1500 h of testing at 45 °C and a relative humidity of 45%.

**Figure 11:**
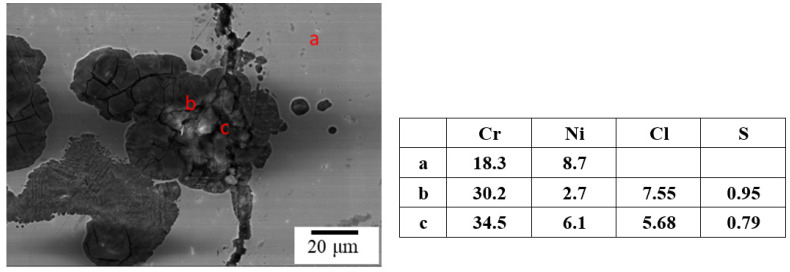
The inclusions in the cracks were examined by the EDX technique.

**Figure 12:**
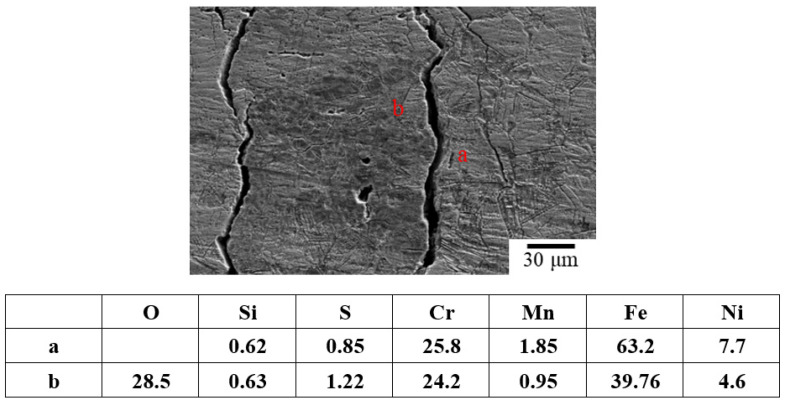
The oxide films at the specimen surface around the cracks were examined by the EDX technique.

Figure 6 and Figure 7 reveal the crevice corrosion specimens after being tested under tensile stress at 45 °C and 45% relative humidity. The specimens were sprayed with 4 g/m^2^ and 0.1 g/m^2^ of sea salt on their surfaces, respectively. It was expected that an increase in the tensile stress would increase the possibility of SCC, in terms of crack numbers and crack size. For the specimen deposited with 4 g/m^2^ of salt, cracks were found on all the specimens, and more cracks developed as the applied stress was increased; for the salt deposit of 0.1 g/m^2^, SCC was not observed at the low stress of 200 MPa for the 1000 h crevice corrosion test. As the stress was increased, small cracks were found on the specimens subjected to the applied stress of 400 and 300 MPa, as shown in Figure 8 and Figure 9, respectively. For the stress level of 400 MPa, cracks were obviously observed for the specimens with high deposits of salt. For the specimens sprayed with a salt deposit of 0.1 g/m^2^, one crack was observed on the surface. For the test at the stress level of 300 MPa, the crack initiated from the surface defect. The length of the crack was less than 400 μm, as shown in Figure 9, and the depth was about 240 μm, as shown in Figure 10. Therefore, the crack growth rate was about 0.16 µm/hour, approximately 1.4 mm/year. The surface defects of the specimen were confirmed to be sulfide by EDX, as shown in Figure 11 and Figure 12. The sulfide near the surface delaminates the passive film and increases the acidity, owing to the dissolution of sulfide [19,20]. As sea salt contains chloride and thiosulphate, the EDX results revealed the presence of sulfide and chloride, which produced acidic conditions. They supplement the stainless steel matrix in the process of corrosion. The chromium content increased on the corrosion surface, owing to the fact that the constant hydrolysis of chromium hydroxide is significantly different from that of iron and the other metal hydroxide [21]. The chloride and sulfide ions migrated into the crevice and conversely other cations not involved in the hydrolysis reactions migrated into the bulk solution environment. As a consequence, the EDX results presented more chromium at the surrounding fissure than the matrix, and a small amount of sulfide and chloride remained at the fissure.

### 3.3. The EBSD Analysis

**Figure 13:**
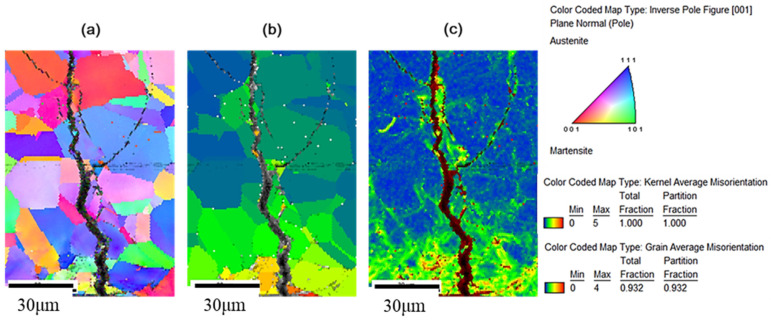
(**a**) Crystal orientation, (**b**) GAM, and (**c**) KAM maps by EBSD analysis of the specimen with 4 g/m^2^ of sea salt deposited on the surface after testing at 300 MPa tensile stress for 1500 h at 45 °C and 45% relative humidity.

**Figure 14:**
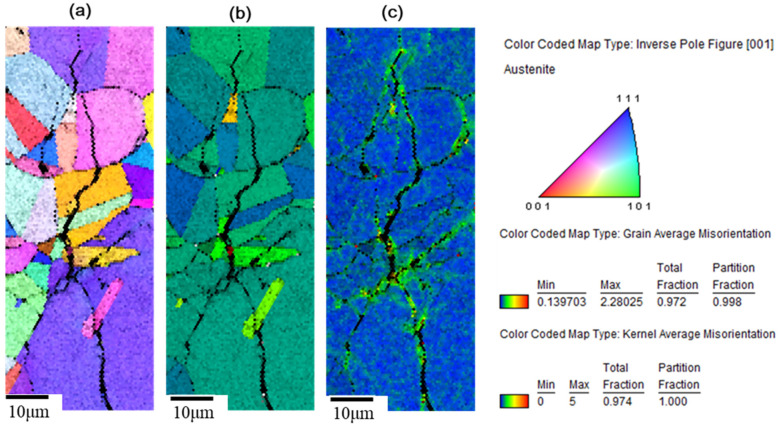
(**a**) Crystal orientation, (**b**) GAM, and (**c**) KAM maps by EBSD analysis of the specimen, as shown in Figure 11.

Figure 13 and Figure 14 show the crystal orientation map of inverse pole figure (IPF) coloring, the grain average misorientation (GAM) map, and the kernel average misorientation (KAM) map by electron backscatter diffraction (EBSD) analysis. Crystal orientation maps show that the cracks are mainly transgranular. The GAM maps illustrate the localized deformation of the grain. This suggests that the strain energy is stored in the grains containing cracks. In addition, the surface shows a higher strain and it is possible that the strain gradually decreases with increasing the distance from the specimen’s surface, as shown in Figure 13. As the crack develops, as shown in Figure 14, the local electrochemical activity within the grain dominates the crack growth process, and the cracks branch inside the grain and then discontinue. The crack tip absorbs hydrogen and enhances the local plasticity of the grain, which increases the resistance of the dislocation movement [10]. Furthermore, discontinuous micro-cracks enhance the effective stress intensity and crack propagation [22]. In Figure 13 and Figure 14, the kernel average misorientation (KAM) maps show that the strain occurs within a short distance around cracks. In addition, the GAM maps show that the cracks are mainly transgranular, with a small portion of cracks along the grain boundary.

The hydrogen-enhanced localized plasticity mechanism and adsorption-induced dislocation emission were proposed [9,10,15]. The results of this study supplement the mechanism of hydrogen embrittlement that suggests that the deformation occurs within the grain containing a crack tip, since hydrogen is generated at crack tips in a moist environment. In addition, the mechanism for intergranular cracking might be the formation of martensite on the grain boundary, while transgranular cracking takes place by propagating cracks nucleated at the slip steps by dissolution [12]. As shown on the KAM maps, a higher strain exists on the surface, which opts to form martensite on the surface [16]; the small portion of intergranular cracking mainly occurred at the surface due to grain boundary martensite induced by hydrogen embrittlement. As the crack developed, the transgranular cracking became more vigorous due to the path corrosion that nucleated at the slip steps by dissolution [12], or by the absorption of hydrogen into the austenite [18]. As shown in Figure 10 and Figure 14, a heavy attack along the slip lines nearby the cracks, which is evidence that heavy plastic deformation developed in this region.

## 4. Conclusions

In the present study, the crevice corrosion behavior of 304L stainless steel was studied under a combination of test environments. The test results show that humidity is an important factor affecting the corrosion rate. For a lengthy corrosion test time of 5000 h, the corroded area of the specimen exposed to 70% humidity was three times higher than those exposed to 45% humidity. For the specimens with 0.1 g/m^2^ of sea salt on the surface, SCC susceptibility was low and the pitting corrosion mostly lead to the growth of a single pit. The sea salt containing chloride and thiosulphate produced acidic conditions, which supplemented the stainless steel matrix to corrode and caused the chromium content to increase on the corrosion surface, owing to the constant hydrolysis of chromium hydroxide, which is significantly different from that of iron and the other metal hydroxide. In addition, the surface defects of sulfide from the sea salt prompt the initiation of SCC. For the specimens with 0.1 g/m^2^ of sea salt deposited on the surface subjected to tensile force, the crack growth rate was approximately 1.4 mm/year at the stress of 300 MPa in a crevice corrosion environment of 45 °C and 45% relative humidity.

The crack tip enhanced the local plasticity of the grain and increased the deformation of the grains containing the crack tip, causing the cracks to develop. The small portion of intergranular cracking mainly occurred on the surface, which was attributable to the existed strain and the hydrogen embrittlement caused by strain-induced formation of martensite along grain boundaries. As the crack developed, transgranular cracking was vigorous due to path corrosion that nucleated at the slip steps.

## Figures and Tables

**Table 1:** The compositions of the base metal of the 304L stainless steel plates (wt%).

Element	C	Si	Cr	Ni	Mn	S	Fe
wt%	0.017	0.45	18	9	1.54	0.029	Bal

**Table 2:** The chemical composition of the simulated sea salt (wt%).

Composition	NaCl_2_	MgCl_2_·6H_2_O	Na_2_SO_4_	CaCl_2_	KCl	NaHCO_3_	KBr	H_3_BO_3_	SrCl_2_·6H_2_O	NaF
%	58.49	26.46	9.75	2.765	1.645	0.477	0.238	0.071	0.095	0.007
